# Letter to the Editor (reply to Souty C et al.): The causes of long‐term trends in the epidemiology of influenza

**DOI:** 10.1111/irv.12636

**Published:** 2019-02-27

**Authors:** Saverio Caini, John Paget

**Affiliations:** ^1^ Netherlands Institute for Health Services Research (NIVEL) Utrecht The Netherlands

To the Editor,

We read Souty et al's^1^ paper on the declining size of influenza epidemics in France during 1984‐2017 with interest. The authors acknowledge that similar trends have been observed in other countries as well, both in Europe (eg, the Netherlands and the United Kingdom) and in outside (New Zealand), and discuss what factor(s) could have driven the observed trend. We agree with the authors' opinion that the increased vaccination coverage is unlikely to have played a major role. In fact, vaccine uptake is low in France (as elsewhere in Europe) in the age groups that experience the highest incidence rates, that is, children, adolescents, and young adults. Consistent with this point, Sprujit et al^2^ failed to find a convincing association between influenza vaccination coverage and influenza‐like illness in fourteen European countries.

Hygiene improvements and changes in the biological properties of circulating viruses are invoked as alternative, potential key drivers of the observed trends. While we agree that these factors may have made an important contribution, the authors may have overlooked a further possible cause, which is the changes in climate that have occurred in Europe over the past 40 years.^3^ There is ample epidemiological evidence that supports the notion that the occurrence of influenza epidemics is driven by climatic parameters, in particular absolute humidity and temperature in temperate climate countries.^4^ Under cold‐dry conditions, which are common in temperate countries during the winter months, virus survival and host susceptibility are enhanced, thus favoring the transmission of influenza viruses and the onset of epidemics.^5^ Researchers have long expressed concern that ongoing climate change may have modified (and continue to do so in the future) the epidemiology of viral respiratory infections, including influenza, for instance by modifying the timing, size, and duration of epidemics.^6^, ^7^

We recently studied the association between climatic parameters and weekly influenza‐like illness (ILI) activity in the Netherlands during 1970‐2016, and reported two main results.^8^ First, weekly absolute humidity was inversely associated with 1‐ and 2‐week lagged ILI incidence; second, the seasonal average absolute humidity was correlated, in an ecological analysis, with the seasonal average ILI incidence (similar results were obtained using temperature instead of absolute humidity, although to a slightly lesser degree). The relationship between climatic parameters and ILI incidence was therefore observed both within and between seasons. In particular, we found that season average ILI incidence rate in the Netherlands has started to rise again since the early 2000s after three decades of marked decline, and this has coincided with a trend reversal of seasonal average absolute humidity, which took place approximately in the same years. The latter finding is of particular interest because several of the factors (other than climate change) that can affect the transmission of respiratory viruses and whose changes are often hypothesized as possible drivers of long‐term trends in influenza incidence (eg, hygienic standards, social contact patterns, household size, urbanization level, patterns of mobility, and others) do not appear to be able to explain why ILI incidence has started to rise again in the last fifteen years in the Netherlands. Ecological studies are never conclusive and require confirmation in analytical studies, yet we believe that climate change should always be considered when looking into, or speculating about, causes of long‐term trends in the incidence of respiratory viral diseases.

## CONFLICT OF INTEREST

The authors declare they have no conflict of interests to disclose.

## AUTHORS' CONTRIBUTIONS

SC conceived the article and wrote the first draft. SC and JP reviewed and edited the article.

## References

[1] Souty C , Amoros P , Falchi A , et al. Influenza epidemics observed in primary care from 1984 to 2017 in France: a decrease of epidemic size over time. Influenza Other Respir Viruses. 2019;13(2):148–157. 10.1111/irv.12620 PMC6379635 30428158

[2] Spruijt IT , de Lange MM , Dijkstra F , Donker GA , van der Hoek W . Long‐term correlation between influenza vaccination coverage and incidence of influenza‐like illness in 14 European countries. PLoS ONE. 2016;11(9):e0163508. 27684558 10.1371/journal.pone.0163508 PMC5042488

[3] European Environment Agency . Global and European temperature 2018. https://www.eea.europa.eu/data-and-maps/indicators/global-and-european-temperature-8/assessment. Accessed January 15, 2019.

[4] Tamerius JD , Shaman J , Alonso WJ , et al. Environmental predictors of seasonal influenza epidemics across temperate and tropical climates. PLoS Pathog. 2013;9(3):e1003194. 23505366 10.1371/journal.ppat.1003194 PMC3591336

[5] Shaman J , Pitzer VE , Viboud C , Grenfell BT , Lipsitch M . Absolute humidity and the seasonal onset of influenza in the continental United States. PLoS Biol. 2010;8(2)e1000316. 20186267 10.1371/journal.pbio.1000316 PMC2826374

[6] Towers S , Chowell G , Hameed R , et al. Climate change and influenza: the likelihood of early and severe influenza seasons following warmer than average winters. PLoS Curr. 2013;5. 10.1371/currents.flu.3679b56a3a5313dc7c043fb944c6f138 PMC3770759 24045424

[7] Mirsaeidi M , Motahari H , Taghizadeh Khamesi M , Sharifi A , Campos M , Schraufnagel DE . Climate change and respiratory infections. Ann Am Thorac Soc. 2016;13(8):1223‐1230. 27300144 10.1513/AnnalsATS.201511-729PS

[8] Caini S , Spreeuwenberg P , Donker G , Korevaar J , Paget J . Climatic factors and long‐term trends of influenza‐like illness rates in The Netherlands, 1970–2016. Environ Res. 2018;167:307‐313. 30081307 10.1016/j.envres.2018.07.035

